# Developing an approach for assigning GRADE levels in a systematic overview of reviews of diagnostic test accuracy using general principles identified from current GRADE guidelines: A case study

**DOI:** 10.1017/rsm.2025.10047

**Published:** 2025-10-13

**Authors:** Andrew Dullea, Lydia O’Sullivan, Kirsty K. O’Brien, Patricia Harrington, Marie Carrigan, Susan Ahern, Maeve McGarry, Karen Cardwell, Michelle O’Neill, Kieran A. Walsh, Barbara Clyne, Susan M. Smith, Mairin Ryan

**Affiliations:** 1 Discipline of Public Health and Primary Care, School of Medicine, Trinity College Dublin, The University of Dublin, Dublin, Ireland; 2 Health Technology Assessment Directorate, Health Information and Quality Authority, Cork, Ireland; 3 Health Research Board-Trials Methodology Research Network, College of Medicine, Nursing and Health Sciences, University of Galway, Galway, Ireland; 4 School of Pharmacy, University College Cork, Cork, Ireland; 5 Department of Public Health and Epidemiology, Royal College of Surgeons in Ireland (RCSI), University of Medicine and Health Sciences, Dublin, Ireland; 6 Department of Pharmacology and Therapeutics, Trinity College Dublin, The University of Dublin, Dublin, Ireland **Permanent Address of A.D.:** Health Information and Quality Authority, George’s Court, George’s Lane, Smithfield, Dublin 7, D07 E98Y, Ireland.

**Keywords:** GRADE, overview, research synthesis, review of reviews, systematic review, umbrella review

## Abstract

Existing guidelines on overviews of reviews and umbrella reviews recommend an assessment of the certainty of evidence, but provide limited guidance on ‘how to’ apply the Grading of Recommendations Assessment, Development, and Evaluation (GRADE) to such a complex evidence synthesis. We share our experience of developing a ‘general principles’ approach to applying GRADE to a complex overview of reviews. The approach was developed in an iterative and exploratory manner during the planning and conduct of an overview of reviews of a novel molecular imaging technique for the staging of prostate cancer, involving a formal review by a group of 11 methodologists/health services researchers. This approach was developed during the evidence synthesis process, piloted, and then applied to our ongoing overview of reviews. A ‘general principles’ approach of applying the domains of GRADE to an overview of reviews and arriving at an overall summary judgement for each outcome is presented. Our approach details additional factors to consider, including addressing both the primary study risk of bias as assessed by the included reviews and the risk of bias of the systematic reviews themselves, as well as the statistical heterogeneity observed in meta-analyses conducted within the included reviews. Our approach distilled key principles from the relevant GRADE guidelines and allowed us to apply GRADE to a complex body of evidence in a consistent and transparent way. The approach taken and the methods used to develop our approach may inform researchers working on overviews of reviews, umbrella reviews, or future methodological guidelines.

## Highlights

### What is already known?

Existing guidelines on overviews of reviews and umbrella reviews recommend an assessment of the certainty of evidence, but provide limited guidance on ‘how to’ apply the Grading of Recommendations Assessment, Development, and Evaluation (GRADE) to such a complex evidence synthesis.

### What is new?

We developed an approach to applying GRADE to a complex overview of reviews. This methodological approach builds on previous case studies in the area.

### Potential impact for RSM readers

Details of our experience contribute to known gaps in existing guidelines. The approach and its development may be of interest to other researchers.

## 1 Introduction

The Grading of Recommendations, Assessment, Development, and Evaluation (GRADE) framework is a widely adopted method for developing and presenting summaries of evidence.^1^ It aims to provide a systematic, transparent approach for assessing the certainty of evidence to support clinical practice recommendations, and was originally developed with systematic reviews of effectiveness (as well as clinical guidelines and health technology assessments) in mind. However, there has been growing interest in the application of GRADE to other forms of evidence synthesis, such as overviews of reviews or umbrella reviews, which are conceptually very similar methodologies that involve synthesising existing systematic reviews.^2^ ^–^ ^4^

Guidance for conducting umbrella reviews or overviews of reviews from the Johanna Briggs Institute (JBI) and the Cochrane Collaboration recommends that an assessment of the certainty of the evidence should be undertaken.^3^ ^,^ ^5^ The JBI guidance suggests that reviewers apply the principles of GRADE to each outcome or phenomenon of interest, whereas the Cochrane guidance recommends using the information from the included systematic reviews to apply GRADE when it is not possible to extract GRADE assessments from the systematic reviews themselves. However, neither Cochrane nor JBI provides specific details on the steps required when applying GRADE to such complex evidence syntheses.

A scoping review by Gates et al.^6^ found that while most available guidance (77%) on overviews of reviews recommended using the GRADE approach, formal guidance on how to apply GRADE in the context of an overview of reviews was not yet available. For example, there is no guidance on how to handle the separate but related issues of the risk of bias in both the primary studies and in the systematic reviews when making a GRADE judgement on how the risk of bias might affect the overall certainty of the evidence. In the absence of such guidance, researchers may be dissuaded from applying GRADE or assessing the certainty of evidence. This may, in turn, result in overviews of reviews or umbrella reviews that lack a key step in aiding the translation of evidence into recommendations or decisions.

Pollock et al.^4^ ^,^ ^7^ have described their experience of developing an algorithm for applying GRADE to overviews of reviews, which they subsequently applied to a Cochrane overview on interventions for improving upper limb function after stroke. Their approach focused on the development of cut-offs and ‘concrete rules’, which reflected careful consideration of the type of evidence included in their overview of reviews. We build upon this dialogue on GRADE for complex evidence syntheses and share our experience of developing a ‘general principles’ approach for applying GRADE to overviews of reviews and umbrella reviews. Herein, we aim to describe the approach taken to apply GRADE to an overview of reviews on a novel molecular imaging technique in the staging of prostate cancer, which then helped inform a national health policy decision on the justification of this new practice.^8^ ^,^ ^9^ The overview of reviews to which this approach was applied is available via open access from Seminars in Nuclear Medicine.^9^

## 2 Materials and methods

**Figure 1:**
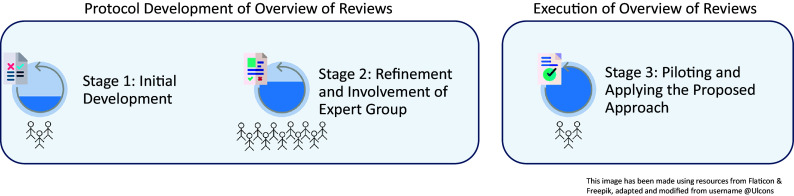
Schematic depicting the development of the GRADE approach.

Methodological development of our approach began during the protocol development for an overview of reviews of the diagnostic accuracy of ^18^F-prostate specific membrane antigen positron emission tomography/computed tomography (PET/CT) staging in prostate cancer. This overview of reviews was pre-registered, and the protocol, report, and publication have been subsequently published.^9^ ^–^ ^12^ It may serve as a useful practical example for readers considering the approach specified herein. Our process for developing these methods was exploratory and was revised iteratively following discussion and piloting.^4^ We developed the approach in three distinct stages: initial development and piloting by a core team of researchers (A.D., L.O.S., and K.K.O.B.), involvement of a wider network of methodologists and health services researchers (S.S., P.H., K.W., B.C., S.A., K.C., M.M.G., and M.O.N.), followed by subsequent revisions, and finally further piloting (on two outcomes) and refinement prior to applying the agreed methods. The plan for developing our approach was set out *a priori* as part of the project plan for our overview of reviews. Each stage was completed consecutively during the evidence synthesis process, as illustrated in Figure 1. At the end of each stage, the approach was refined to ensure that it was transparent, consistent, and true to the overarching GRADE guidelines we identified as relevant to our overview of reviews. The lead researcher (A.D.) kept detailed meeting notes and the version history of documents in order to capture the iterative development of the approach taken.

### 2.1 Stage 1: Initial development

In order to inform the approach taken, we began by identifying relevant methodological studies and guidance. Our objective was not to conduct multiple systematic scoping reviews of methodological guidance for GRADE, overviews of reviews, and umbrella reviews. We instead informed our approach by consulting with our experienced network of methodologists and conducting a brief search of the literature via MEDLINE. Influential references included both the JBI and Cochrane guidance, the GRADE handbook, two recent scoping reviews, and papers detailing approaches taken to date by others in applying GRADE to overviews of reviews or umbrella reviews.^2^ ^–^ ^6^ ^,^ ^13^ ^–^ ^15^ Guidelines 1, 3, 16, 21 (parts 1 and 2), 22, and 36 of the GRADE series were agreed by the team as the most relevant to our specific overview of reviews of diagnostic test accuracy (DTA).^16^ ^–^ ^22^ However, it is conceivable that a similar method could be used to apply the general principles of GRADE to other outcomes. Consideration was given to a short article published by Murad et al.^23^ on rating the certainty in evidence in the absence of pooled effect estimates; however, its focus on systematic reviews of interventions limited its applicability to our overview of reviews. We also searched the reference lists from two key scoping reviews on methodological approaches to overviews of reviews and umbrella reviews.^2^ ^,^ ^6^ From these materials, we extracted key principles that addressed five domains for downgrading (risk of bias, inconsistency, indirectness, imprecision, and publication bias) and two domains for upgrading (test outcome relations and residual bias or confounding) with respect to test accuracy outcomes. Per GRADE guidance 31, there are just two domains for upgrading comparative test accuracy outcomes compared to the three domains typically seen in systematic reviews of interventions.^24^ These key principles were added to a working document; the team then added additional considerations to account for biases and issues that could be introduced by the systematic reviews. We documented a number of important considerations during this stage, which are detailed in our results.

### 2.2 Stage 2: Further refinement and involvement of a wider expert group

We circulated the proposed approach in a Microsoft Word document to a wider group of 11 multidisciplinary methodologists affiliated with or working at the Royal College of Surgeons in Ireland, University College Cork, Trinity College Dublin, and the Health Information and Quality Authority. A meeting was then held via Microsoft Teams to facilitate feedback, conduct a formal review, and reach consensus. A consensus was unanimously reached for each section by actively seeking and addressing any objections.

### 2.3 Stage 3: Applying the proposed approach to the overview

Additional revisions from the wider group were incorporated, and the approach was initially piloted by A.D. on 2 of the 26 DTA outcomes of interest. The core team conducted the overview of reviews and then met to agree upon the GRADE assessments for the 26 DTA outcomes. We discussed each domain, the overall number of downgrades or possible upgrades, and the certainty of evidence assessed by the GRADE approach (high, moderate, low, and very low) for each outcome until consensus was reached. We recorded all summary judgements and judgements for each domain on an Excel spreadsheet.^9^

## 3 Results

The approach developed and applied to our overview of reviews is outlined below. The core team of three health services researchers reached consensus on each judgement without dissenting views. Each domain was judged to be not serious, serious, or very serious for each outcome. A GRADE Excel template for the overview of reviews is available from the online repository (https://osf.io/fpzxd/), and we have provided an example illustrating the application of our approach to outcomes in this overview of reviews in Table 1.^10^

**Table 1:** Example applying GRADE to an overview of reviews previously published in seminars of nuclear medicine.

			Risk of bias						Grading		
	No.	No.	Primary	Systematic							
	primary	systematic	study	review				Publication	Initial	No. of	Final
Outcomes	studies	reviews^a^	(QUADAS-2)	(ROBIS)	Inconsistency	Indirectness	Imprecision	bias	GRADE	downgrades	GRADES
*Per-patient level*											
Sensitivity	10	4	Serious^b^	Very serious^c^	Not serious	Not serious	Not serious	Serious^d^	High	1^e^	Moderate
Specificity	7	4	Serious^b^	Very serious^c^	Serious^f^	Not serious	Serious^g^	Serious^d^	High	2	Low
Accuracy	0	0	—	—	—	—	—	—	N/A	—	N/A
*Per-lymph node (N-stage)*											
Sensitivity	0	0	—	—	—	—	—	—	N/A	—	N/A
Specificity	0	0	—	—	—	—	—	—	N/A	—	N/A
Accuracy	0	0	—	—	—	—	—	—	N/A	—	N/A
*Per-lesion (M-stage)*											
Sensitivity	6	3	Serious^b^	Serious^h^	Very serious^i^	Not serious	Serious^g^	Serious^d^	High	3	Very low
Specificity	5	3	Serious^b^	Serious^h^	Serious^j^	Not serious	Not serious	Serious^d^	High	2	Low
Accuracy	1	2	Not serious	Serious^h^	Serious^f^	Not serious	Very serious^k^	Not serious	High	2	Low
*Other outcomes*											
Dose	0	0	—	—	—	—	—	—	N/A	—	N/A
Adverse Events	0	0	—	—	—	—	—	—	N/A	—	N/A

Abbreviations: GRADE, Grading of Recommendations, Assessment, Development, and Evaluations; N/A, not applicable; QUADAS, quality assessment of diagnostic accuracy studies; ROBIS, risk of bias tool for systematic reviews.

a Some of these reviews did not include the specified outcome in their review; however, data from all possible reviews were used to gather details on the primary study and its risk of bias. Hence, the number of reviews cited here may differ from the number of reviews referred to in Section 3 of our overview of reviews.

b Some of the QUADAS-2 domains within these primary studies were at an unclear to high risk, but many were still low risk.

c Most review studies had a high risk of bias. Most domains within these reviews were at unclear or high risk of bias.

d The rationale for a serious or very serious judgement on the ROBIS was considered; however, it was felt that there were still residual issues with the publication bias, comprehensiveness of the search, search strategy, or inclusion and exclusion criteria.

e Large and consistent effect sizes were taken into account when considering how many levels to downgrade by.

f Considerably large inconsistency and variation in point estimates across studies, particularly within the meta-analysis performed by Yang et al.,^43^ or too few estimates from too few studies.

g Large confidence intervals of primary studies and/or the overall pooled estimate provided by Yang et al.^43^

h Many reviews had an overall unclear or high risk of bias. Many of the domains within these reviews were at an unclear to high risk.

i Highly inconsistent results and point estimates. We also considered the statistical heterogeneity to be serious.

j Variation in point estimates across studies and some statistical heterogeneity of concern.

k No confidence intervals, far too few events, or suspected far too few events, where the number of events was not reported within the systematic review.

### 3.1 Overall approach to applying GRADE

Informed by the literature on GRADE and overviews of reviews, the core team considered DTA outcomes to be initially high certainty, which could then be downgraded based on five domains, and potentially upgraded thereafter based on two further domains.^2^ ^–^ ^6^ ^,^ ^13^ ^–^ ^22^ As per the GRADE guidelines and in keeping with the assumptions of our overview of reviews, cross-sectional or cohort studies involving patients with diagnostic uncertainty and direct comparisons of test results with an appropriate reference standard started as high certainty but could be rated down to moderate, low, or very low certainty depending on other factors.^17^ ^,^ ^20^ ^,^ ^25^ ^,^ ^26^ As no other study designs were included in our overview of reviews, all outcomes started as high certainty.

During Stage 2, the wider group decided to:

- use terminology consistent with the standard GRADE terminology to avoid conceptual confusion, as previously indicated elsewhere,^27^
- avoid ‘double penalising’ the same outcome for similar reasons, for example, where the ROBIS assessment already took into account the publication bias in the systematic review, and
- use a general principles approach to arrive at an overall global judgement, rather than implementing a scoring system or concrete rules.

We discussed the application of general principles and an overall summary judgement for each outcome during both Stage 1 and Stage 2 of developing our approach. We agreed to apply this approach because when assigning penalties or scores for each domain, outcomes repeatedly appeared to accrue multiple penalties, which resulted in outcomes being consistently downgraded to ‘very low’ certainty. The core team felt that this was not reflective of the true certainty of the evidence, and that the use of penalties or scores for each domain led to poor discriminatory capacity between ‘high’ and ‘very low’ certainty evidence.

During Stage 3, the core team noted that some reviews included in the overview of reviews did not report confidence intervals for some estimates. Systematic reviews were cross-referenced in the first instance to ensure that the confidence intervals were not reported elsewhere. In accordance with the GRADE guidance and guidance on overviews of reviews, and given that the review was our unit of inclusion, we did not refer to the primary studies but instead used the number of events or sample size to inform the judgement of imprecision.

The core team faced challenges in defining the ‘number of systematic reviews’ for each outcome in the GRADE table. For example, four primary studies reported on one particular outcome of interest, and these four studies were included in five systematic reviews. However, some of these systematic reviews did not report on that particular outcome but instead focused on other outcomes reported in the primary studies.

Even if a systematic review did not report on the specific outcome, it still conducted a risk of bias assessment on the primary study, which is relevant. The team agreed that all reviews that provided a risk of bias assessment for a study should be considered when evaluating the risk of bias for the primary studies, regardless of whether some of these had excluded the exact outcome we were assessing. This was clearly denoted in our tables (see Table 1).

### 3.2 Approach to downgrading domains

#### 3.2.1 Risk of bias

In judging the severity of bias in both the primary studies and systematic reviews, we looked for general patterns of concern across all the QUADAS-2 (for the primary studies) and ROBIS domains (for the included reviews) where the risk of bias was deemed unclear or high.^28^ ^,^ ^29^ Additionally, we looked for systematic patterns within specific domains for unclear or high-risk biases. We also took into account the overall risk of bias presented by the systematic reviews.

The core team felt that there was a need to consider *both* the biases in the primary studies and the systematic reviews. If the team only assessed the risk of bias of the systematic reviews, then important biases arising from the design, conduct, and reporting of the primary studies could be missed. Similarly, if only the risk of bias of the primary studies was considered, then there is a risk of ignoring important biases introduced by the design, conduct, and reporting of the systematic reviews.

To address these issues, given the focus of the overview of reviews, we first extracted the QUADAS-2 assessments (a tool to assess the quality of DTA studies) reported by systematic review authors to judge the risk of bias of the primary studies. Thereafter, we assessed the risk of bias of the systematic reviews using the risk of bias in systematic reviews (ROBIS) tool. Both assessments were used to inform judgements on their risk of bias.

Due to overlap between systematic reviews, multiple QUADAS-2 assessments were available for some primary studies. We initially proposed using a conservative approach, where we only considered the assessments that rated the study to be at a higher risk of bias relative to other assessments by other systematic reviewers. However, we later decided to use all assessments and acknowledge discrepant judgements where present, as the amount of discrepant judgements was relatively small and easy to visualise on our summary table of risk of bias assessments (see the Supplementary Material of our overview).^9^ However, we acknowledge that this may not be possible in other overviews of reviews where there is large interrater variability for risk of bias assessments.

Although our overview focused on QUADAS-2 data, this approach is likely generalisable to other risk of bias assessments. The developers of QUADAS-2 highlight that the assessments are not intended to be used to generate a summative ‘quality score’; hence, when judging the risk of bias of primary studies, we looked for general issues across domains and for consistent issues in any of the specific domains assessed.

#### 3.2.2 Inconsistency

In keeping with the GRADE guidance on inconsistency, we first judged this domain based on the variability of the primary study effect (i.e., the variability of point estimates across studies reported in the various systematic reviews) and the extent to which confidence intervals had minimal or no overlap. However, statistical heterogeneity from meta-analyses also played a role in influencing our judgements on inconsistency as recommended in the GRADE handbook.^13^

To facilitate judgements in this domain, forest plots were developed for each important outcome with more than two results. After first reviewing the forest plots themselves, we considered the seriousness of the inconsistency based on whether the *I* ^2^ was substantial (50%–90%) or considerable (75%–100%) as specified by *Section 9.5.2 of the Cochrane Handbook* and *Section 3.3.10.2 of the JBI Manual for Evidence Synthesis*.^5^ ^,^ ^30^ ^,^ ^31^ Contextual consideration was given to the possibility that a high *I* ^2^ may be observed where there are large studies with minimal overlap, but otherwise very similar point estimates. It is important to highlight that as with traditional systematic reviews, the *I* ^2^ value is only one factor, which may help provide insight into concerns regarding inconsistency. GRADE guidance 36 clarifies the role of *I* ^2^ in this domain, and that GRADE inconsistency is addressing variability in study results rather than variability in other areas such as study design.^21^

Pooled estimates from meta-analyses were not readily available for the specific research questions posed in this overview of reviews, and we often judged inconsistency on the variability in the primary study results, as suggested in GRADE guidance 36 and the GRADE handbook, rather than based on a pooled estimate.^13^ ^,^ ^21^ Similar to judgements on *I* ^2^, there should be an explanation for the observed variability, and if no plausible explanation was present, we considered downgrading. The decision to treat inconsistency as ‘serious’ or ‘very serious’ depended on the extent of the variability observed. Estimates of inconsistency for small single studies were considered ‘very serious’, as although there is no evident heterogeneity, the estimates for that outcome have not been validated elsewhere. However, where we downgraded outcomes reported within only one small study for inconsistency, we did not also downgrade for imprecision to avoid double-penalising.

#### 3.2.3 Indirectness

The core team noted that according to GRADE 21 part 1, some researchers may make indirect comparisons based on separate studies in which each test was compared against a reference standard and then downgraded for indirectness. We were aware from initial scoping that there was a lack of studies that compared the PET/CT to standard imaging investigations. We also knew there was a lack of ‘test-and-treat’ RCTs related to our research question. We agreed *a priori* and during protocol development that DTA would be considered a surrogate for the impact of testing on patient important outcomes, and test accuracy studies would start as high quality. In line with the GRADE guidelines, we were explicit in describing that we only considered the certainty of the evidence for DTA studies and would not downgrade for the indirectness of this surrogate outcome.

However, when moving from evidence to decision-making using our modified Evidence to Decision (EtD) making framework, the two-step process of linking evidence between different studies and different diagnostic tests (e.g., evidence on MRI vs. evidence on PET-CT) was considered in the judgements made.^18^ ^,^ ^22^

As we included ‘high-risk patients’ and ‘patients with biochemical recurrence’ using any definition for both groups, we did not downgrade for indirectness, as definitions vary between guidelines.^32^ However, we did factor into our considerations downgrading in situations where outcomes may not be generalisable to all patients with either high-risk or biochemical recurrent prostate cancer—for example, when outcome data were only available for patients with certain histopathological features that may not represent all patients with high-risk prostate cancer or biochemical recurrence. This consideration is generalisable to broader types of overviews of reviews or umbrella reviews; however, we acknowledge that many considerations in this domain, by necessity, were specifically focused on DTA.

#### 3.2.4 Imprecision

We assessed imprecision by considering the number of events, as well as the width and overlap of confidence intervals, as suggested by the GRADE guidance for test accuracy.^4^ ^,^ ^18^ ^,^ ^19^ Where confidence intervals were not reported, and/or where the number of events or lesions (i.e., the denominator in some cases) was not reported, we judged the imprecision to be either ‘serious’ or ‘very serious’ depending on the extent to which this happened in the reported estimates synthesised. To facilitate judgements in this domain, forest plots were developed for each important outcome with more than two results.

#### 3.2.5 Publication bias

Our approach to assessing publication bias was largely based on the comprehensiveness and quality of the systematic review literature search, the inclusion of grey literature or trial registry data, the presence of only studies that produce precise estimates of high accuracy despite small sample sizes, and the influence of industry funding. The decision to assess publication bias under these factors was influenced by the GRADE guidance and the GRADE checklist.^33^ To a lesser extent, we also considered the results of Deeks’ tests or Trim and Fill methods where the systematic review question was similar or identical to the overview of reviews question. Results from funnel plots (e.g., Egger’s or Begg’s tests) in systematic reviews of test accuracy are likely to result in downgrading for publication bias more frequently than appropriate; hence, these had little influence on the core team’s judgement.^19^ Although non-inferiority test accuracy studies may suffer from a unique publication bias situation due to their ability to assess statistical significance with Bayesian methods, it was not possible to assess this phenomenon in the grading of the certainty of the evidence within this overview.^34^

As many of these factors are also included in the ROBIS assessment, care was taken not to inadvertently ‘double penalise’ reviews under risk of bias and publication bias for the same issue. Where an outcome was already downgraded due to risk of bias in the systematic reviews’ search methods, our judgements were largely limited to whether there was the presence of only small studies that produced precise estimates for high accuracy despite the small sample sizes, and the possible role of industry funding.

### 3.3 Approach to upgrading domains

In relation to upgrades, two domains for upgrades (rather than the three typically seen when GRADE is used for reviews of therapeutic interventions) were used. As all our included outcomes started as ‘high’, these domains could only mitigate potential downgrades rather than upgrading a given outcome further.

#### 3.3.1 Test outcome relations and large effect estimates

As noted in the GRADE guidelines, the certainty in test accuracy may increase if summary receiver operator characteristic curves show a clear and consistent sensitivity-specificity relationship.^19^ As we did not produce pooled meta-analysed results ourselves, we looked for large and consistent effect sizes across the body of evidence. In our overview of reviews, there was only one instance in which we needed to consider the large and consistent effect size. In this scenario, we had initially considered downgrading by two levels due to the seriousness of issues in other domains; however, taking into account the large effect estimates observed, we decided to downgrade by only one level instead of two. A footnote was included in our table to reflect this. This domain is similar to the domains of ‘large magnitude of effect’ and ‘dose–response gradient’ seen in other parts of the GRADE series.

#### 3.3.2 Residual plausible bias or confounding

As noted in the GRADE guidelines, the certainty in test accuracy may increase if there is very high accuracy in the presence of minimal opposing residual confounding. If there were instances where it was felt that the magnitude of effect was decreased by the confounding present, we considered upgrading. There was no instance in our overview of reviews where we felt that downgrades could be ameliorated by possible residual bias or confounding.

## 4 Discussion

In this paper, we aimed to develop an approach broadly consistent with guidance from JBI and Cochrane, whereby we apply the principles of GRADE in a general manner to an overview of reviews, and arrive at overall summary judgements for each GRADE domain and for each outcome.^3^ ^,^ ^5^ ^,^ ^9^ While there are challenges to applying GRADE to complex evidence syntheses, such as overviews of reviews, the use of a consistent and clearly documented approach, such as the one presented, can support transparency and confidence in the conclusions that are drawn. This general principles approach may be of interest to those conducting either overviews of reviews or umbrella reviews due to the similarity of their methods and the fact that the unit of inclusion is often—but not always—limited to systematic reviews.^35^ Although the aspects of our approach are specific to DTA outcomes, the methods we used to distil key principles may have relevance to overviews of reviews and umbrella reviews in general.

Although GRADE is suggested by JBI and Cochrane,^3^ ^,^ ^5^ it is not the only method for assessing the certainty of the evidence. One scoping review indicated that most umbrella reviews use some type of ‘credibility assessment’ to determine the certainty of evidence instead.^2^ Such alternative approaches have been proposed elsewhere^36^; however, they have been criticised in favour of GRADE largely due to the issues with the overreliance on *p*-values to assess the clinical relevance of findings, omission of important domains covered by GRADE (such as risk of bias), and use of arbitrary cut-offs.^37^ Additionally, there is no consensus that such an alternative criterion is the method of choice.

Other authors have previously attempted to apply GRADE to overviews of reviews. One such example conducted GRADE assessments on each individual outcome for *each* systematic review, which essentially meant performing the GRADE process for each systematic review included in the overview.^15^ However, depending on the overview of reviews and umbrella review research question, this may often lead to a large summary of findings tables that include a number of identical or very similar outcomes with possible conflicting judgements, making the interpretation and communication of findings more difficult. Research questions for these types of complex evidence syntheses may also be broad or open-ended. While this was not a major issue in our case study, others may find that this leads to difficulties, particularly where the PICOs of the included systematic reviews are quite dissimilar. This, again, may lead to a large summary of findings tables that contain multiple similar outcomes and possibly conflicting judgements.

The methods proposed by Pollock et al.^4^ present another option to authors of overviews of reviews, with the establishment of clear, concrete, and predefined rules. However, complex evidence bases present reviewers with many difficulties, as noted by Berkan et al.^38^ In our overview, this complexity arose from the lack of GRADE assessments within the systematic reviews themselves, specific considerations for DTA outcomes, lack of relevant meta-analyses, difficulty in deciding appropriate cut-offs, and lack of high-quality RCT data. Our overview of reviews also included many reviews with unclear or high risk of bias, which would not have been considered with Pollock et al.’s approach, making it difficult to adopt or modify for our purposes. Instead, by reverting back to the original guidelines and identifying the key principles, we were able to GRADE the certainty of the evidence with reasonable confidence.

Merits of our work include the involvement of a multi-disciplinary team and the extensive methodological experience of the individuals involved. This collaborative effort enabled the use of a GRADE EtD framework and subsequently facilitated national health policy decision-making.^12^ However, a number of important limitations of our approach exist. First, it is important to note that no structured methods (e.g., Delphi) were employed to develop consensus on the approach taken, and the collaborators on this project were all based in Ireland. Methods used to develop our approach were exploratory and pragmatic, and were chosen based on an institutional need to address the gap in the current guidance. Another important limitation arose from the fact that very few pooled estimates from meta-analyses were included in our overview of reviews. The use of a narrative synthesis reported in line with the synthesis without meta-analysis reporting guidelines and the PRIOR statement was instead used to enable health policy decision-making. However, if there is a need to focus on pooled estimates for systematic reviews, our proposed approach may require further modification.^14^ ^,^ ^39^ Future researchers may wish to examine the between-review heterogeneity of pooled results, rather than or in addition to the heterogeneity between primary study estimates extracted from the narrative synthesis.

In this paper, we have presented one possible approach that allows for both transparency and reproducibility, which may be suitable for use in other overviews of reviews or bodies of evidence. It is an approach that may be of interest to methodologists and researchers from different disciplines interested in GRADE and overviews of reviews or umbrella reviews, particularly given the growing role of such syntheses in informing policy, guidelines, and overall decision-making inside and outside of clinical practice.^40^ ^,^ ^41^ While the key principles identified here are specific to DTA studies, similar principles could be distilled from other articles in the GRADE series or from the GRADE working group to design an approach more appropriate to other evidence bases, which might include time-to-event outcomes, for example.^1^ ^,^ ^42^

## 5 Conclusion

Our approach distilled key principles from the relevant GRADE guidelines and allowed us to apply GRADE to a complex body of evidence in a consistent and transparent way. The approach taken and the methods used to develop our approach may be of relevance to researchers working on overviews of reviews, umbrella reviews, or future methodological guidelines.

## Data Availability

The data that support the findings of this study are openly available from the Open Science Framework at https://doi.org/10.17605/OSF.IO/QMEZ5 and from our previously published overview of reviews at https://doi.org/10.1053/j.semnuclmed.2024.05.003.
